# Combination Measles-Mumps-Rubella-Varicella Vaccine in Healthy Children: A Systematic Review and Meta-analysis of Immunogenicity and Safety

**DOI:** 10.1097/MD.0000000000001721

**Published:** 2015-11-06

**Authors:** Shu-Juan Ma, Xing Li, Yi-Quan Xiong, A.-.ling Yao, Qing Chen

**Affiliations:** From the Department of Epidemiology, School of Public Health and Tropical Medicine, Southern Medical University (S-JM, Y-QX, QC); Guangdong Provincial Institute of Public Health, Guangdong Provincial Center for Disease Control and Prevention (XL); and Department of Health Statistics, School of Public Health and Tropical Medicine, Southern Medical University, Guangzhou, China (A-LY).

## Abstract

A combined measles-mumps-rubella-varicella (MMRV) vaccine is expected to facilitate universal immunization against these 4 diseases. This study was undertaken to synthesize current research findings of the immunogenicity and safety of MMRV in healthy children.

We searched PubMed, Embase, BIOSIS Previews, Web of Science, Cochrane Library, and other databases through September 9, 2014. Eligible randomized controlled trials (RCTs) were selected and collected independently by 2 reviewers. Meta-analysis was conducted using Stata 12.0 and RevMan 5.3.

Twenty-four RCTs were included in qualitative synthesis. Nineteen RCTs compared single MMRV dose with measles-mumps-rubella vaccine with or without varicella vaccine (MMR + V/MMR). Similar seroconversion rates of these 4 viruses were found between comparison groups. There were comparable geometric mean titers (GMTs) against mumps and varicella viruses between MMRV group and MMR + V/MMR group. MMRV group achieved enhanced immune response to measles component, with GMT ratio of 1.66 (95% confidence interval [CI] 1.48, 1.86; *P* < 0.001) for MMRV versus MMR and 1.62 (95% CI 1.51, 1.70; *P* < 0.001) for MMRV versus MMR + V. Meanwhile, immune response to rubella component in MMRV group was slightly reduced, GMT ratios were 0.81 (95% CI 0.78, 0.85; *P* < 0.001) and 0.79 (95% CI 0.76, 0.83; *P* < 0.001), respectively. Well tolerated safety profiles were demonstrated except higher incidence of fever (relative risks 1.12–1.60) and measles/rubella-like rash (relative risks 1.44–1.45) in MMRV groups.

MMRV had comparable immunogenicity and overall safety profiles to MMR + V/MMR in healthy children based on current evidence.

## INTRODUCTION

Combination measles-mumps-rubella-varicella (MMRV) vaccine was originally designed as an alternative to separate measles-mumps-rubella (MMR) vaccine and varicella (V) vaccine, based on similar vaccination schedules and good concomitant safety profiles.^1–3^ Two MMRV vaccines have been available since mid-2000: ProQuad (Merck & Co., Inc, West Point, PA; Merck) and Priorix-Tetra (GlaxoSmithKline Biologicals, Rixensart, Belgium; GSK). Both have been widely used in USA, Australia, Canada, and many European countries. The MMRV vaccine was developed based on the existing MMR and varicella vaccines.^4,5^ Various formulations and schedules of the vaccine were investigated during the development process to obtain acceptable immunogenicity and safety profiles. Licensed ProQuad and Priorix-Tetra have different measles virus strains (Edmonston stain and Schwarz stain, respectively) with the same titer (≥10^3.0^ tissue culture 50% infective dose, TCID_50_). Mumps virus strain in Priorix-Tetra (RIT 4385 strain, titer ≥10^4.4^ TCID_50_) is derived from what is used in ProQuad (Jeryl Lynn strain, titer ≥10^4.3^ TCID_50_). ProQuad and Priorix-Tetra have same rubella virus strain (Wistar RA 27/3 strain) and titer (≥10^3.0^ TCID_50_). They also have same varicella virus strain (Oka strain), but with different titers (≥10^3.99^and ≥10^3.3^ plaque-forming units, respectively).

The MMRV vaccine is expected to offer several benefits: simplifying immunization delivery; increasing compliance with immunization; decreasing cumulative exposure to additives; and reducing healthcare costs. ^5–7^ Up to date, although several reviews^5,7–10^ focused on the immunogenicity and safety of ProQuad and/or Priorix-Tetra have been published, we lack a systematic understanding of MMRV vaccine. This study was conducted as a meta-analysis of clinical trials to investigate the immunogenicity and safety of 1 and 2-dose vaccination courses of MMRV vaccine in healthy children.

## METHODS

### Eligibility Criteria

Eligible study designs were randomized controlled trials (RCTs) comparing MMRV-vaccinated children (MMRV group) with MMR-vaccinated (MMR group) or MMR + varicella vaccine coadministered children (MMR + V group). The population of interest were healthy children aged 0 to 6 years, irrespective of sex and ethnic origin. The intervention was any unlicensed or licensed MMRV regardless of administration route, dosage, and schedule. Outcomes related to immunogenicity and safety of vaccines had been reported.

### Literature Search

We searched PubMed, Embase, BIOSIS Previews (1994–2013), Web of Science, Cochrane Library, National Institutes of Health database (clinicaltrials.gov), GSK Clinical Trials (gsk-clinicalstudyregister.com), and MERCK Clinical Trials (merck.com/clinical-trials) from the earliest date available through September 9, 2014. We used key words or subject headings for (“measles” and “mumps” and “rubella” and “varicella”) or (“measles mumps rubella varicella” or “MMRV”) in combination with “vaccine,” including their common synonyms. There were no language restrictions. We screened bibliographies of selected original studies, review articles, and relevant conference abstracts, and contacted corresponding authors for missing or unpublished data.

### Study Selection

Citations were merged together in Endnote, version X6, to facilitate management. Two reviewers independently applied the inclusion criteria to all retrieved articles and records of clinical trials in an unblinded standardized manner, evaluated by title, abstract, and full text. Disagreements between reviewers were resolved by consensus.

### Data Collection

Data were mainly obtained from published articles. Unpublished clinical trials served as a supplement. Priority was given to data published in case of subtle discrepancy. For each of the eligible study, information of general study, study population, treatment, and outcomes of immunogenicity and/or safety was selectivity extracted onto piloted structured forms independently by 2 reviewers.

### Quality Assessment

The internal validity of meta-analysis included clinical trials assessed using the Jadad score,^11^ applying a score from 0 (very poor quality) to 5 (rigorous), based on the following items: randomization and adequate performance of the randomization procedure; double-blind and the adequate performance; and description of withdrawals and dropouts. Studies were graded on an ordinal scoring scale with higher scores representing studies of higher quality.

### Statistical Analysis

Analysis of immunogenicity was performed mainly on according-to-protocol cohorts. Seroconversion or seroprotection rate and geometric mean titers (GMTs) for antibodies against each vaccine component after each dose were calculated with 95% confidence intervals (CIs) in each study. With respect to GMT, a log10 transformation was performed for the GMT to ensure normality, and the standard deviation (SD) was calculated from 95% CI using the calculator in RevMan 5.3 software (Cochrane Collaboration) to get completed continuous data. Analysis of safety was performed on per total vaccinated cohort. The incidences of solicited and/or unsolicited local, general symptoms, and adverse events were calculated.

Relative risks (RRs) for binary results and weighted mean difference (WMD) for continuous findings were calculated. Between-study heterogeneity was assessed by using Cochrane Q statistic and quantified by estimated *I*^2^. A Mantel–Haenszel fixed-effects model (M-H, fixed) for binary data and an inverse variance fixed-effects model (IV, fixed) for continuous data were used to calculate when the test for heterogeneity was not statistically significant (*P* > 0.10); otherwise, DerSimonian–Laird random-effects models (DL, random) were employed.^12–14^ All statistical tests were 2-sided and considered significant when the *P* value was ≤0.05. We also performed sensitivity analyses to evaluate whether any single study dominated the results of meta-analyses. Finally, publication bias was assessed by quantitative Begg funnel plots for outcomes reported by the above 10 trials.^15^ Statistical analyses were conducted using Stata 12.0 (StataCorp, College Station, Texas, USA) and RevMan 5.3.

## RESULTS

### Description of Studies Included

**FIGURE 1:**
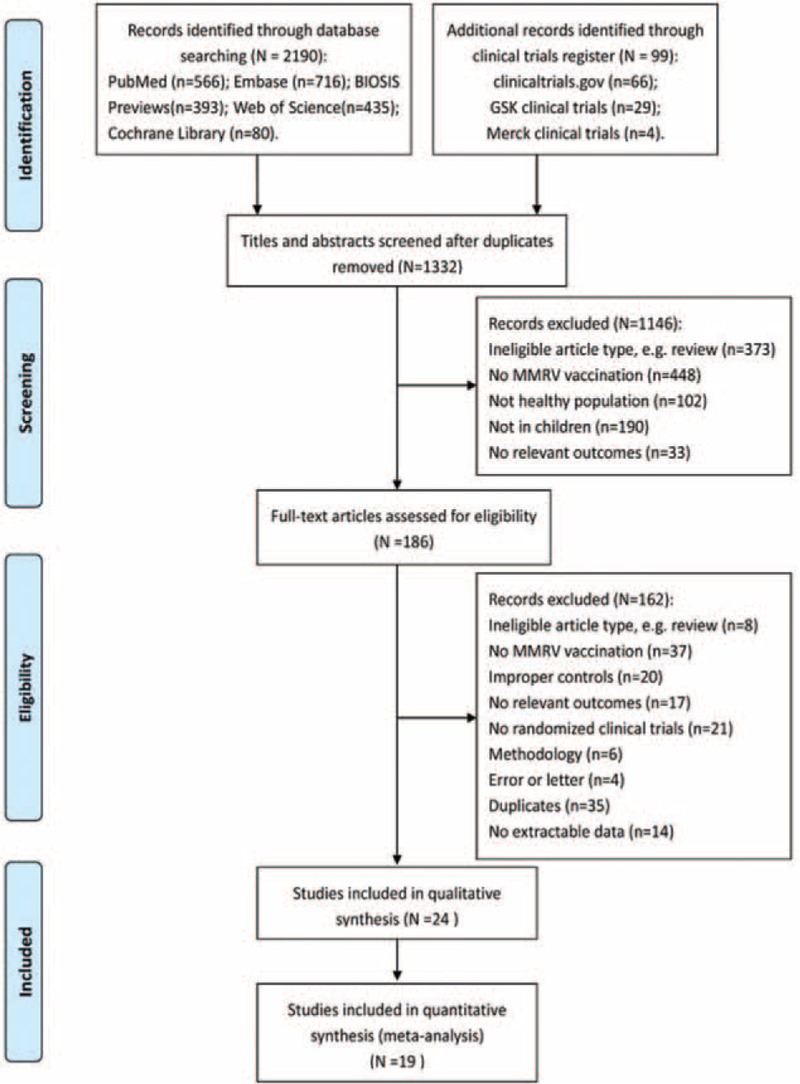
PRISMA flow diagram of study selection. GSK = GlaxoSmithKline Biologicals, Merck = Merck & Co., MMRV = measles-mumps-rubella-varicella vaccine.

A total of 2190 studies and 99 clinical trial records were originally identified through online searching. The selection process is shown in Figure 1. Twenty-four articles published/unpublished RCTs were included in the review and summarized in Table 1 .^16–39^ Considering the different controlled groups and vaccination intervals, only immunogenicity and safety profiles after first MMRV dose were compared and meta-analyzed. Of the 24 selected RCTs, 5 RCTs^24,27,28,37,39^ were not included in the meta-analysis, because MMRV vaccine in these studies was given as second dose following a first dose of MMR or MMR + V vaccine. Other 19 RCTs involving 21098 subjects were included in the quantitative analysis. Comparisons were conducted between MMRV group and MMR + V or MMR group (MMR + V/MMR group). Fifteen RCTs^16–23,25,26,30,32,34–36^ compared the immunogenicity and safety between MMRV group and MMR + V group (MMRV vs MMR + V), and 6 RCTs^19,29,31,33,36,38^ compared those between MMRV group and MMR group (MMRV vs MMR). Between-study heterogeneity was explored by the MMRV vaccine manufacturer (Merck-MMRV and GSK-MMRV) mainly. Furthermore, 9 RCTs ^20–22,25,26,29,31,33,34^ involving 11527 subjects of 2-dose schedule of MMRV were included in the qualitative analysis with various controlled groups. As scores of the Jadad scale were equivalent to 2 or 3 in most clinical trials, there was not a stratified analysis by ranking for little difference of the quality between included RCTs.

**TABLE 1:**
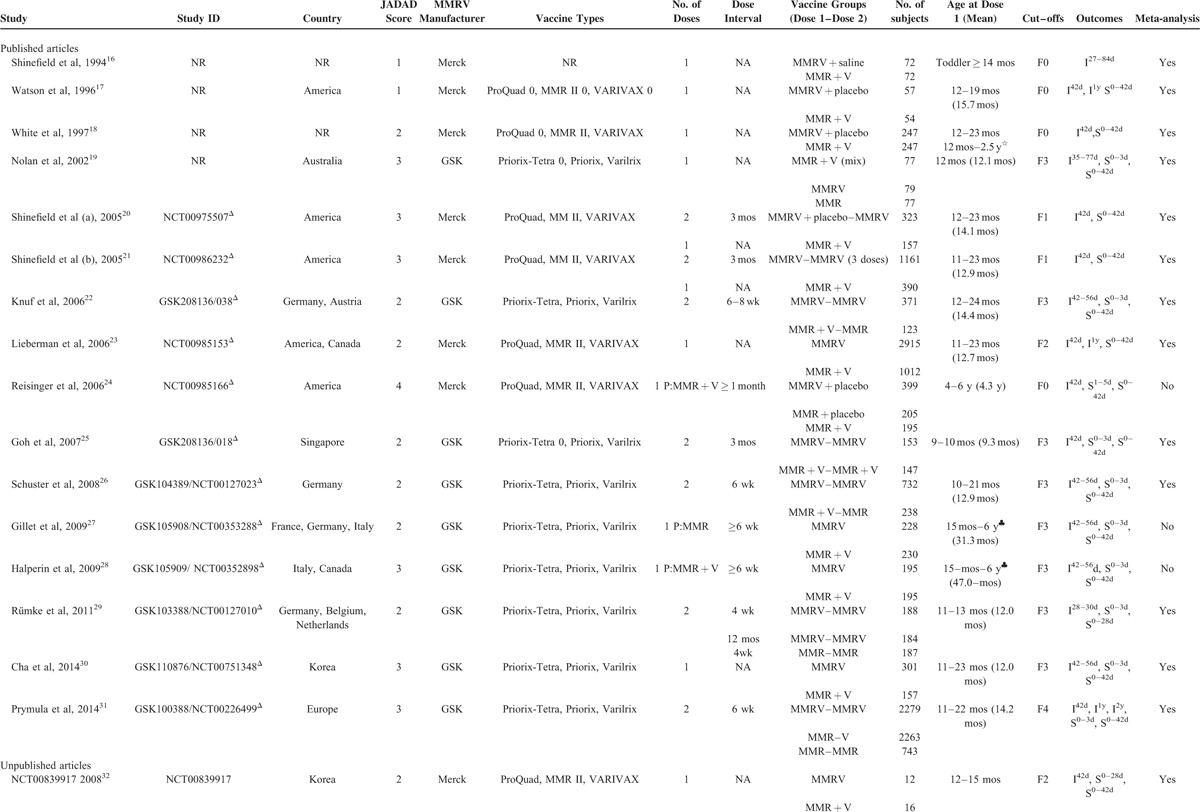
Characteristics of Included Published/Unpublished RCTs

**TABLE 1 (Continued):**
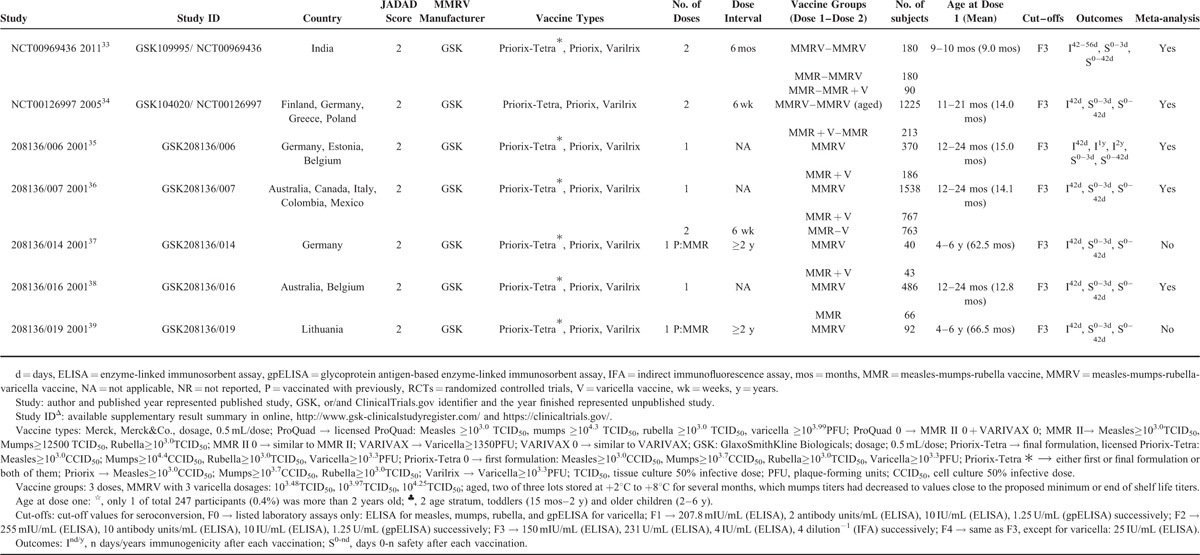
Characteristics of Included Published/Unpublished RCTs

### Single MMRV Dose in Healthy Children Aged 9 to 24 Months

#### Immunogenicity

All included studies reported seroconversion rate as serological response outcome, except for varicella in Merck-MMRV–vaccinated studies, which was seroprotection rate. Seroconversion rate was defined as percent of subjects initially seronegative (with titers ≤ assay cut-offs), who developed postvaccination antibody titers above the assay cut-off levels. Seroprotection rate for varicella was defined as the proportion of subjects who were seronegative at baseline and whose postvaccination titer was ≥5 units/mL detected by the glycoprotein antigen-based enzyme-linked immunosorbent assay. The serological response rates and GMTs for all 4 antigens 27 to 84 days after vaccination were meta-analyzed in studies with comparison groups of MMR + V, whereas 3 antigens (measles, mumps and rubella) for comparison groups of MMR. Considering the different laboratory assays and corresponding cut-offs (Table 1 ), only GSK-MMRV–vaccinated studies with same assays and cut-offs were combined for the analysis of GMT in this article.

#### Measles

**FIGURE 2:**
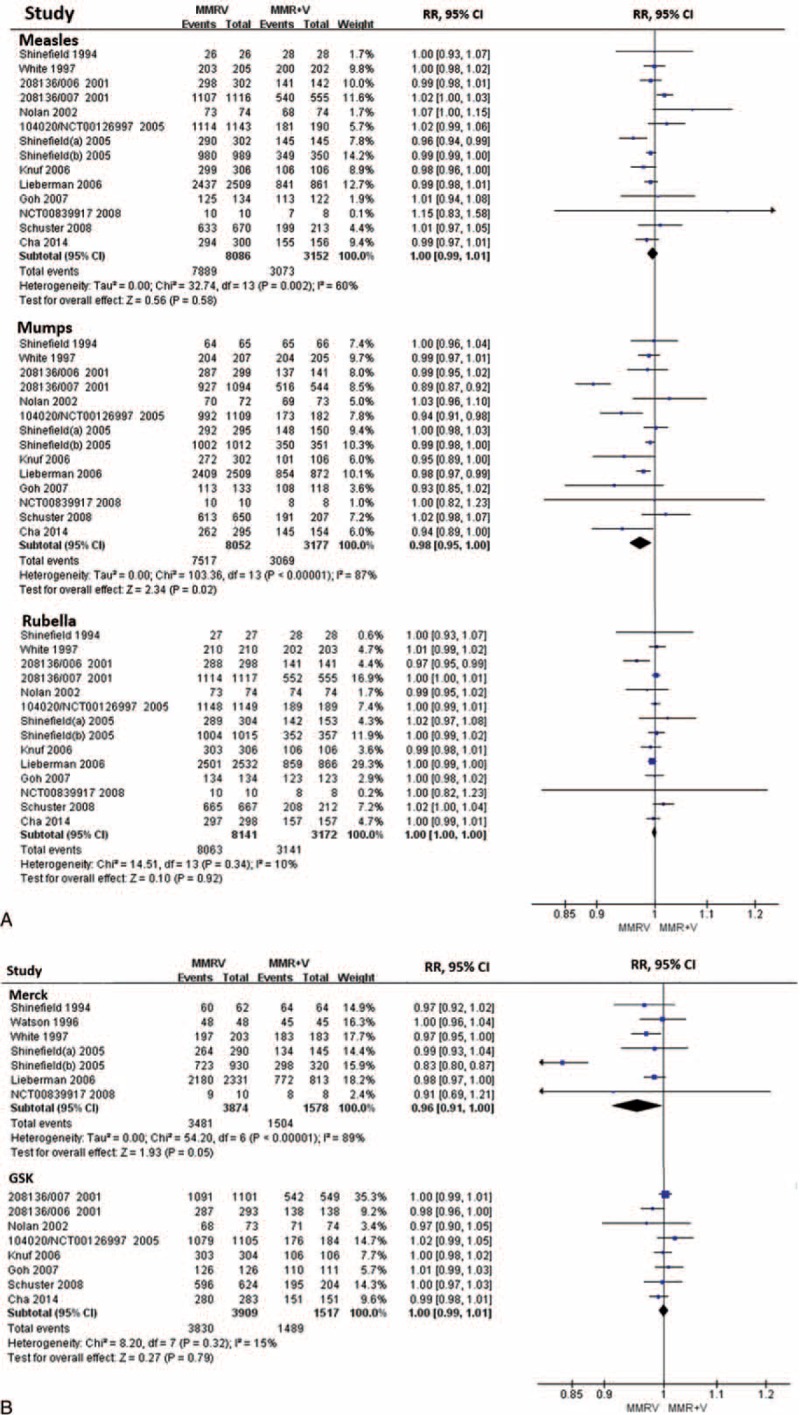
A, Forest plot of seroconversion rates for measles, mumps, and rubella employing 14 RCTs (MMRV vs MMR + V). B, Forest plot of seroconversion/seroprotection rate for varicella employing 15 RCTs (MMRV vs MMR + V). GSK = MMRV in this comparison was manufactured by GlaxoSmithKline Biologicals, Merck = MMRV in this comparison was manufactured by Merck & Co., MMR = measles-mumps-rubella vaccine, MMRV = measles-mumps-rubella-varicella vaccine, RCTs (MMRV vs MMR + V) = randomized controlled trials comparing single MMRV dose with MMR + V coadministered in healthy children, V = varicella vaccine.

**FIGURE 3:**
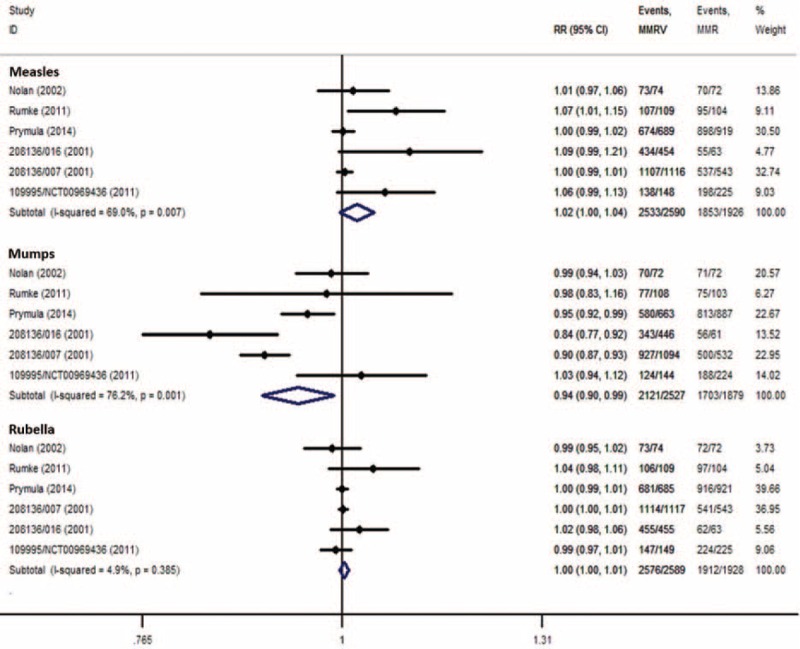
Forest plot of seroconversion rates for measles, mumps, and rubella employing 6 RCTs (MMRV vs MMR). MMR = measles-mumps-rubella vaccine, MMRV = measles-mumps-rubella-varicella vaccine, RCTs (MMRV vs MMR) = randomized controlled trials comparing single MMRV dose with MMR administered in healthy children.

Fourteen RCTs (MMRV vs MMR + V)^16,18–23,25,26,30,32,34–36^ and 6 RCTs (MMRV vs MMR)^19,29,31,33,36,38^ reported seroconversion rate for measles, and were included in the analysis. Seroconversion rates for measles were more than 93.2% in MMRV groups in both comparisons, ranged from 87.5% to 100% in MMR + V groups, and ranged from 88.0% to 98.9% in MMR groups. Pooled RR of the 14 RCTs (MMRV vs MMR + V) was 1.00 (DL, random; 95% CI 0.99, 1.01; *P* = 0.580) with the evidence of heterogeneity as measured by *I*^2^ statistic (*I*^2^ = 60%, *P* = 0.002). Point-estimated RRs ranged from 0.96 to 1.15 (Figure 2A). Similarly, pooled RR of the 6 RCTs (MMRV vs MMR) reported seroconversion rate for measles was 1.02 (DL, random; 95% CI 1.00, 1.04; *P* = 0.092), with considerable heterogeneity among studies (*I*^2^ = 69%, *P* = 0.007). Point-estimated RRs ranged from 1.00 to 1.09 (Fig. 3). There was no statistical difference between MMRV group and MMR + V/MMR group.

**FIGURE 4:**
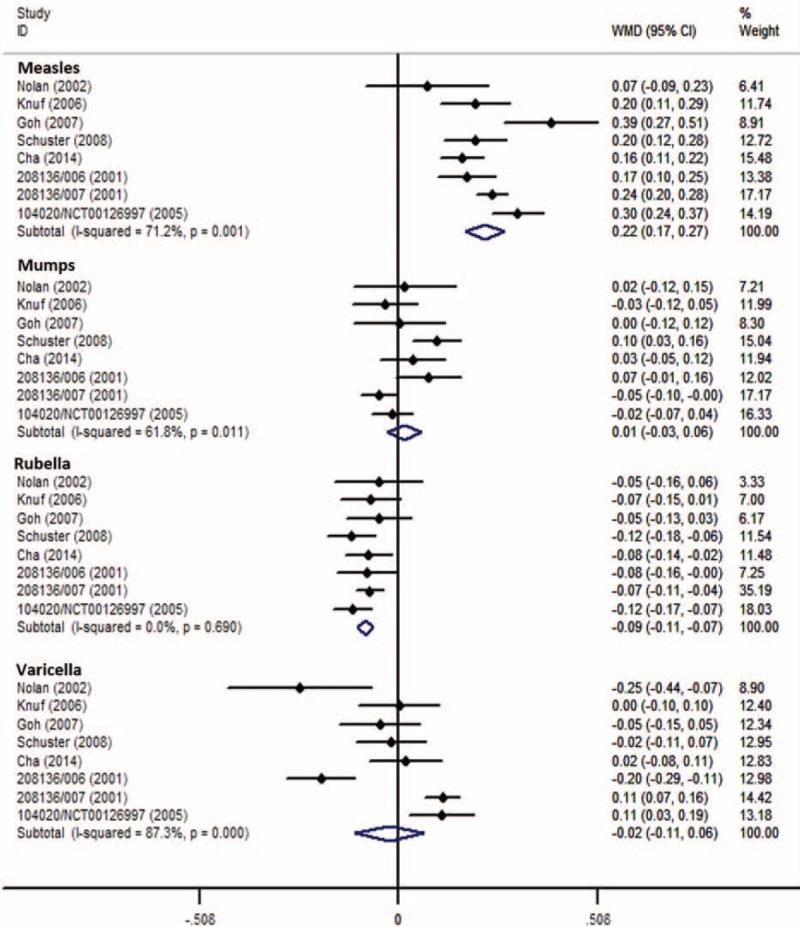
Forest plot of antimeasles, antimumps, antirubella, and antivaricella GMTs employing 8 RCTs (MMRV vs MMR + V). GMTs = geometric mean titers, MMR = measles-mumps-rubella vaccine, MMRV = measles-mumps-rubella-varicella vaccine, RCTs (MMRV vs MMR + V) = randomized controlled trials comparing single MMRV dose with MMR + V coadministered in healthy children, V = varicella vaccine.

**FIGURE 5:**
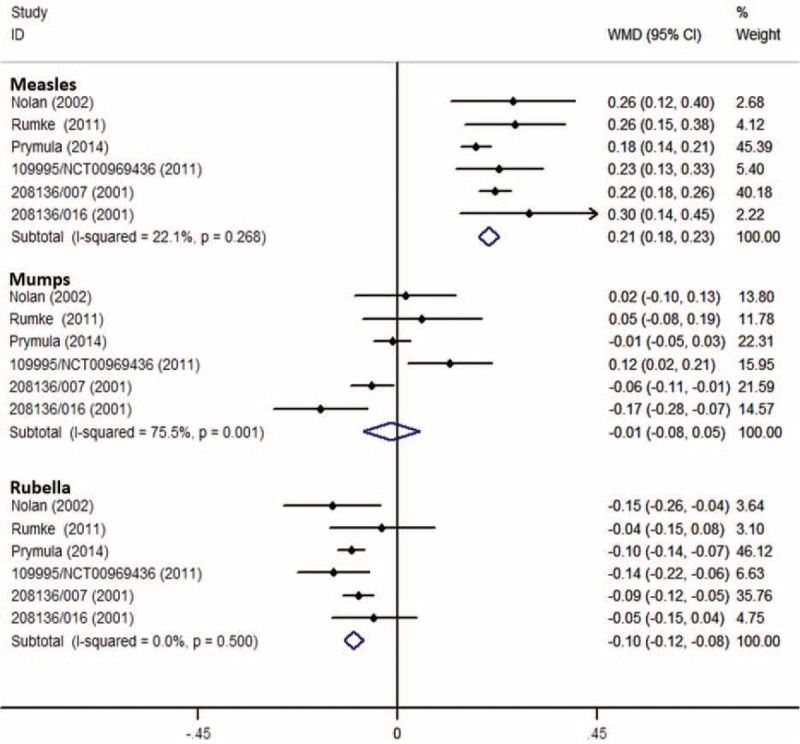
Forest plot of antimeasles, antimumps, and antirubella GMTs employing 6 RCTs (MMRV vs MMR). GMTs = geometric mean titers, MMR = measles-mumps-rubella vaccine, MMRV = measles-mumps-rubella-varicella vaccine, RCTs (MMRV vs MMR) = randomized controlled trials comparing single MMRV dose with MMR administered in healthy children.

Eight RCTs (MMRV vs MMR + V)^19,22,25,26,30,34–36^ with same assay and cut-off value for measles were included to analyze antimeasles GMT. Overall analysis had a high heterogeneity (*I*^2^ = 71%), the corresponding WMD was 0.22 (DL, random; 95% CI 0.17, 0.27; *P* < 0.001), and point-estimated WMDs ranged from 0.07 to 0.39 (Figure 4). Six RCTs (MMRV vs MMR)^19,29,31,33,36,38^ had the same analysis, getting a WMD of 0.21 (IV, fixed; 95% CI 0.18, 0.23; *P* < 0.001), with *I*^2^ of 22% (Figure 5). Sensitivity analyses showed that results were robust by removing 1 or 2 studies with higher weights. All the results suggested that antimeasles GMT was higher in MMRV group than those in MMR + V/MMR group with a significant difference; the GMT ratios were 1.66 (95% CI 1.48, 1.86; *P* < 0.001) and 1.62 (95% CI 1.51, 1.70; *P* < 0.001), respectively.

#### Mumps

Fourteen RCTs (MMRV vs MMR + V)^16,18–23,25,26,30,32,34–36^ reported seroconversion rate for mumps, in which the rates ranged from 84.7% to 100% in MMRV groups and from 91.5% to 100% in MMR + V groups. Pooled RR was 0.98 (DL, random; 95% CI 0.95, 1.00; *P* = 0.020; *I*^2^ = 87%) (Fig. 2A). Six RCTs (MMRV vs MMR)^19,29,31,33,36,38^ reported seroconversion rate for mumps; the rates ranged from 71.3% to 97.2% in MMRV groups and from 72.8% to 98.6% in MMR groups. Pooled RR was 0.94 (DL, random; 95% CI 0.90, 0.99; *P* = 0.017; *I*^2^ = 76%) (Fig. 3). Subgroup analysis found that significant difference and heterogeneity mainly came from GSK-MMRV–vaccinated studies. Three studies^26,35,36^ in MMRV versus MMR + V comparison and 2 studies^35,36^ in MMRV versus MMR comparison had greater influence to the pooled estimated effect based on sensitivity analyses. After the exclusion of them, corresponding pooled RRs were 0.95 (M-H, fixed; 95% CI 0.92, 0.97; *P* < 0.001) and 0.97 (M-H, fixed; 95% CI 0.94, 1.00; *P* = 0.047), respectively.

For antimumps GMT, 8 RCTs (MMRV vs MMR + V)^19,22,25,26,30,34–36^ and 6 RCTs (MMRV vs MMR)^19,29,31,33,36,38^ with same assay and cut-off value for mumps were included, respectively (Figs. 4 and 5). All the pooled analyses suggested that there was no significant difference for antimumps GMT between MMRV group and MMR + V/MMR group.

#### Rubella

Fourteen RCTs (MMRV vs MMR + V)^16,18–23,25,26,30,32,34–36^ reporting seroconversion rate for rubella were included, whose rates were all above 95.1% in MMRV groups and above 92.8% in MMR + V groups. In 6 RCTs (MMRV vs MMR),^19,29,31,33,36,38^ seroconversion rates for rubella were all above 97.2% in MMRV groups and above 93.2% in MMR groups. The meta-analyses showed no significant difference in seroconversion rate for rubella between MMRV groups and MMR + V/MMR groups (Figs. 2A and 3).

Additionally, 8 RCTs (MMRV vs MMR + V)^19,22,25,26,30,34–36^ and 6 RCTs (MMRV vs MMR)^19,29,31,33,36,38^ with same assay and cut-off value for rubella were included in 2 analyses of stratifying results. The corresponding pooled WMDs were −0.09 (IV, fixed; 95% CI −0.11, −0.07; *P* < 0.001) (Fig. 4) and −0.10 (IV, fixed; 95% CI −0.12, −0.08; *P* < 0.001) (Fig. 5), respectively. No evidence of homogeneity (*I*^2^ = 0%) was found. Both results suggested that antirubella GMT was mildly lower in MMRV group than that in MMR + V/MMR group; the GMT ratios were 0.81 (95% CI 0.78, 0.85; *P* < 0.001) and 0.79 (95% CI 0.76, 0.83; *P* < 0.001), respectively.

#### Varicella

There were 15 RCTs (MMRV vs MMR + V),^16–23,25,26,30,32,34–36^ which reported seroconversion rate (GSK-MMRV) or seroprotection rate (Merck-MMRV) for varicella; the rates were above 91% in all groups. The hierarchical analysis presented no significant difference of seroconversion rate between GSK-MMRV and MMR + V (M-H, fixed; RR = 1.00; 95% CI 0.99, 1.01; *P* = 0.790). However, there was a significant difference of seroprotection rate between Merck-MMRV and MMR + V, and pooled RR was 0.96 (DL, random; 95% CI 0.91, 1.00; *P* = 0.050; *I*^2^ = 89%) (Fig. 2B).

Analysis of the log10-transformed antivaricella GMT was also performed between MMRV and MMR + V groups. Overall analysis of 8 RCTs^19,22,25,26,30,34–36^ with same assay and cut-off value for varicella got a pooled WMD of −0.02 (DL, random; 95% CI −0.11, 0.06; *P* = 0.579) with great heterogeneity (*I*^2^ = 87%) (Fig. 4). Result was robust evaluated by sensitivity analysis. No significant difference was found for antivaricella GMT between MMRV group and MMR + V group.

### Safety

#### Solicited Local Symptoms

Eight RCTs (MMRV vs MMR + V)^19,22,25,26,30,34–36^ and 6 RCTs (MMRV vs MMR)^19,29,31,33,36,38^ vaccinated with GSK-MMRV were included to analyze the incidences and intensity of solicited local symptoms within 4 days (days 0–3) after vaccination. The pooled analyses are presented in supplementary Table 1 (http://links.lww.com/MD/A491). No heterogeneity was found in all pooled RRs (*I*^2^ = 0%–24%). No significant differences were observed between MMRV and MMR + V/MMR group in incidences of pain, redness, swelling, and their grade 3 levels after the single dose. Redness was most frequently reported (21.58% and 19.16% for MMRV vs MMR + V; 18.45% and 16.21% for MMRV vs MMR). Incidences of pain and swelling were around and below 10% in all groups, respectively. Grade 3 local reactions were rare (<0.5%) for all the vaccine groups, especially pain.

### Solicited General Symptoms

Solicited general symptoms within 43 days (days 0–42) after vaccination from all the pooled analyses are presented in supplementary Table 1 (http://links.lww.com/MD/A491).

Fever was the most frequently reported solicited general symptom, pooled incidences of fever were around 60% in MMRV groups and 50% in MMR + V/MMR groups. Majority were reported during the first 15 days (days 0–14) follow-up period. Half of the events were considered by the investigator to be related to investigational vaccine. Pooled incidence of grade 3 fever (rectal temperature >39.5°C) during the 43 days after vaccination in these studies was relatively low (around 15% in MMRV groups, 11% in MMR + V group and MMR group). Irrespective of follow-up period, higher incidences of fever were reported in MMRV group than in MMR + V/MMR group (pooled RRs ranged from 1.12 to 1.60). All the analyses showed significant difference between comparison groups, except the analyses of related fever during 15 and 43 days after vaccination in MMRV versus MMR + V comparison.

Rash was the second frequently reported solicited general symptom. Pooled incidences of any rash during the first 43 days of follow-up after vaccination ranged from 10.77% to 19.60% in all groups. About one-third of the events were related to investigational vaccine. However, grade 3 rash was rarely reported. Generalized rash (RR = 1.23; 95% CI 1.07, 1.40; *P* = 0.004) and measles/rubella-like rash (RR = 1.44; 95% CI 1.15, 1.81; *P* = 0.002) were significantly more frequent in MMRV group than MMR + V group. Significant difference of incidence of measles/rubella-like rash was only found between Merck-MMRV group and MMR + V group (M-H, fixed; RR = 1.61; 95% CI 1.16, 2.22; *P* = 0.004). Statistical difference of incidence of varicella-like rash was only found between GSK-MMRV group and MMR + V group (M-H, fixed; RR = 1.86; 95% CI 1.12, 3.07; *P* = 0.020). Both incidences of measles/rubella-like rash and varicella-like rash were significantly higher in MMRV groups than those in MMR groups, pooled RRs were 1.45 (M-H, fixed; 95% CI 1.06, 1.98; *P* = 0.020) and 1.95 (M-H, fixed; 95% CI 1.04, 3.66; *P* = 0.040), respectively.

#### Unsolicited Adverse Events

Fifteen unsolicited adverse events (whether or not considered related to the vaccination studied) were analyzed. Those events had relatively high occurrences (>1%) and were reported by at least 3 studies within 43 days (days 0–42) after vaccination. Upper respiratory infection was reported most commonly (>10%) in both comparisons (Supplementary Table 1, http://links.lww.com/MD/A491). Pooled incidences of irritability, otitis media, diarrhoea, rhinorrhea, cough, vomiting, rhinitis, and pharyngitis were above 5%. There was statistically higher incidence of irritability (M-H, fixed; RR = 1.29; 95% CI 1.09, 1.54; *P* = 0.003) in MMRV group than in MMR + V group. Incidence of pharyngitis was statistically higher (M-H, fixed; RR = 1.37; 95% CI 1.09, 1.72; *P* = 0.008) in MMRV group than in MMR group. The hierarchical analysis by MMRV manufacturer showed a pooled RR of pharyngitis in 4 RCTs (GSK-MMRV vs MMR + V) was 1.28 (M-H, fixed; 95% CI 1.01, 1.62; *P* = 0.040).

#### Serious Adverse Events (SAEs)

Incidences of any serious adverse events (SAEs) were around 1% in all the groups; only about one-tenth of the events were considered to be related to vaccination studied. About half of the related SAEs were febrile seizures. The incidence of related febrile seizure was under 0.8‰ in MMRV groups and under 0.5‰ in MMR + V/MMR groups. No statistical difference was found between groups with no evidence of heterogeneity. No related fatal SAE was reported in any studies included.

### MMRV administered as second dose after a first MMR/MMR + V/MMRV in healthy children

Children in 9 RCTs^20–22,25,26,29,31,33,34^ received 2 doses of MMRV vaccines (Table 1 ). Actually, after administering 2 doses of MMRV in healthy children aged 9 to 24 months with an interval of 4 weeks to 6 months, MMRV showed strong immunogenicity against the 4 diseases. Moreover, MMRV was well tolerated compared with a dose of MMR followed by another dose of MMR,^29,31^ MMR + V, or MMRV,^33^ and a dose of MMR + V followed by another dose of MMR^22,26,34^ or MMR + V.^25^ Second dose of MMRV ensured higher seroconversion rates (95%–100%) and GMTs for all vaccine components compared with the single vaccine dose schedule, especially up to 41.6-fold higher for antivaricella GMT.^33^ Comparison between 2 doses of MMRV and MMR + V showed antivaricella GMT increased 10.4-fold (95% CI 8.65, 12.41) in MMRV group and 5.2-fold (95% CI 4.14, 6.53) in MMR + V group, compared with up to 2.2-fold increases for the GMTs of other 3 virus after the second dose in both groups.^25^ Although 1 or 2 solicited local symptoms (pain, redness, and swelling) were more frequently reported after the second dose of MMRV compared to the first dose in most studies,^22,25,26,29,31,33,34^ incidences of most adverse experiences for each comparison after the second dose were similar among groups.

Additionally, five RCTs suggested that MMRV vaccine seemed to be more immunogenic and well tolerated when given as a second dose after MMR (3 RCTs)^27,37,39^ or MMR + V (2 RCTs)^24,28^ vaccination in children aged 15 months to 6 years (Table 1 ).

### Publication Bias

Publication bias was assessed by quantitative Begg funnel plots for 6 outcomes as the numbers of included studies were more than 10. No significant asymmetry was found.

## DISCUSSION

This systematic review synthesized evidence about immunogenicity and safety of MMRV in 24 studies involving more than 23,000 healthy children. We used systematic strategy and broad search terms in multiple databases and related websites to identify as many published and unpublished clinical trials as possible. GMTs against the 4 diseases were assessed by performing a log10 transformation, to get a more intuitional understanding of the immunogenicity of vaccines. However, considering the different laboratory assays and corresponding cut-offs, only the GSK-MMRV–vaccinated studies with same assays and cut-offs were combined for the analyses of GMTs in this article. Moreover, considering the complex control designs and different intervals between doses, only the immunogenicity and safety of single MMRV dose were meta-analyzed in healthy children aged 9 to 24 months.

For immunogenicity, our results from the analysis of 19 RCTs suggested that single MMRV dose in healthy children aged 9 to 24 months had comparable immunogenicity profiles against these 4 diseases to MMR + V/MMR. There are some exceptions as follows:

1. Antimeasles GMT was significantly higher in MMRV group than that in MMR + V/MMR group, the GMT ratios were 1.66 (95% CI 1.48, 1.86; *P* < 0.001) and 1.62 (95% CI 1.51, 1.70; *P* < 0.001), respectively. The results suggested that receipts of GSK-MMRV were more protected against measles, although the measles virus titers were equivalent in MMRV and MMR manufactured by GSK.
2. Pooled seroconversion rate for mumps in MMRV group was significantly lower by 2% to 6% than those in MMR + V group (RR = 0.98; 95% CI 0.95, 1.00; *P* = 0.020) and MMR group (RR = 0.94; 95% CI 0.90, 0.99; *P* = 0.017). However, there was no significant difference for antimumps GMT between MMRV group and MMR + V/MMR group. The slight lower seroconversion rate for mumps might partly be due to the lower mumps virus titer in the early experimental formulation of GSK-MMRV, which led to inclusion of a higher viral titer of mumps component in the final licensed formulation (Priorix-Tetra)_._^10^
3. Antirubella GMT was lower in MMRV group than that in MMR + V/MMR group, with the GMT ratios of 0.81 (95% CI 0.78, 0.85; *P* < 0.001) and 0.79 (95% CI 0.76, 0.83; *P* < 0.001), respectively. This reflected an about 20% lower postvaccination antirubella GMT after GSK-MMRV. Considering the same rubella virus strains and equal virus titers in MMRV and MMR manufactured by GSK, potency of rubella virus in MMRV might be weakened by the existence of varicella virus.
4. A significant difference of seroprotection rate of varicella was found between Merck-MMRV and MMR + V, with the pooled RR being 0.96 (95% CI 0.91, 1.00; *P* = 0.050). The weak reduction might contribute from the lower varicella virus titer in early formulation of Merck-MMRV in previous studies,^16–18^ which was similar to the titer in licensed varicella vaccine manufactured by Merck. Thus, the higher varicella virus titer in licensed Merck-MMRV was necessary to guarantee strong enough protection. Analyses of the single MMRV dose suggest that further developing of MMRV needs pay more attention to the reduced immune response to mumps and rubella components. Researchers should assess the actual impact and try hard to provide comprehensive protection.

To improve individual protection against the 4 diseases and to have a more rapid impact on outbreaks, a second dose catch-up MMRV vaccination has been recommended.^1,6,40^ Actually, after administering 2 doses of MMRV in healthy children aged 9 to 24 months with an interval ranging from 4 weeks to 6 months, MMRV showed strong immunogenicity against the 4 diseases. Second dose of MMRV ensured higher seroconversion rates (95%–100%) and GMTs for all vaccine components compared with the single vaccine dose schedule, especially for varicella virus. The efficacy of 2 doses of MMRV was also in line with estimates of 2 doses of MMR + V, with higher immune response to the 4 virus components.

For safety, single MMRV dose in healthy children aged 9 to 24 months was generally well tolerated. Significant differences were demonstrated mainly in the comparisons of fever and rash. Fever was the most frequently reported solicited general symptom during the 43 days (days 0–42) of follow-up period in all included studies (pooled incidences were above 52.9% in all groups). Higher incidences of fever were found in MMRV groups compared to MMR + V and MMR groups (RRs ranged from 1.12 to 1.60). Rash was the second frequently reported solicited general symptom. Generalized rash (RR = 1.23; 95% CI 1.07, 1.40; *P* = 0.004) and measles/rubella like rash (RR = 1.44; 95% CI 1.15, 1.81; *P* = 0.002) were significantly more frequent in MMRV group than in MMR + V group. Moreover, measles/rubella-like rash (RR = 1.45; 95% CI 1.06, 1.98; *P* = 0.020) and varicella-like rash (RR = 1.95; 95% CI 1.04, 3.66; *P* = 0.040) were significantly more frequent in MMRV group than in MMR group. The results were consistent with a statistical modeling in a previous review, which indicated that the higher level of measles antibody titer after receipt of MMRV was positively associated with the higher rates of fever and measles-like rashes.^8^

It is known that fever can precipitate febrile seizure in susceptible children aged 6 months to 5 years, especially in the 12 to 23-month age range.^41,42^ The higher fever rate made the incidence of febrile seizure be more concerned. In several postmarketing observational safety surveillance studies,^43–46^ an approximate 2-fold increase in risk for seizure or febrile seizure during 7 to 10 days or 5 to 12 days after vaccination were found among children aged 10 to 24 months, those who received the first dose of MMRV compared with those who received the first dose of MMR administered with or without varicella vaccine. However, no significant difference was found in our results in incidence of vaccine-related febrile seizure during the 43 days postvaccination of MMRV compared with MMR + V/MMR. This might partly be due to the limited population, protocol-specified vaccinated ages, and intervals studied in RCTs. SAEs like febrile seizures that occur too infrequently to detect in RCTs may be identified and further studied through postmarketing restudies based on rigorous prospective study design.

Assessment of cost effectiveness based on a dynamic transmission model showed MMRV would provide more quality-adjusted life-years than MMR, and was cost-saving.^47^ Economic analysis also showed that universal mass vaccination against varicella using MMRV to reduce the disease burden of varicella in Germany would lead to cost-savings from societal as well as from the health system perspective.^40^ Considering the higher risk for seizure or febrile seizure, a model performed by Bauchau et al^48^ suggested that use of MMRV instead of MMR + V might substantially reduce number of hospitalizations despite the observed increased risk of febrile seizure, when MMRV was used as a first dose of measles-containing vaccine, which was one of the trade-offs between the two vaccination schemes. Our review highlights that providers who are considering administering MMRV to children should be concerned with the benefits and risks with parents or caregivers. Decisions should be made by them on a case-by-case basis.

## CONCLUSIONS

In conclusion, this systematic review and meta-analysis showed rigorous evidence that MMRV had comparable immunogenicity and overall safety profiles to MMR administered with or without varicella vaccine.
